# Effects of different concentration of organic and inorganic trace minerals (zinc, selenium, and chromium) supplementation on expression of chTLR4 gene and humoral immune response in broilers

**DOI:** 10.14202/vetworld.2021.1093-1101

**Published:** 2021-05-06

**Authors:** Anand Kumar Jain, Aditya Mishra, Ajit Pratap Singh, Pragati Patel, Amir Amin Sheikh, Tilak Ram Chandraker, Rajesh Vandre

**Affiliations:** 1 Department of Veterinary Physiology and Biochemistry, College of Veterinary Science and Animal Husbandry, Nanaji Deshmukh Veterinary Science University, Jabalpur, Madhya Pradesh, India; 2 Animal Biotechnology Centre, Nanaji Deshmukh Veterinary Science University, Jabalpur, Madhya Pradesh, India; 3 Department of Animal Husbandry, Central Semen Station, District Narshinghpur, Madhya Pradesh, India; 4 Division of Veterinary Physiology and Biochemistry, Faculty of Veterinary Science, Sher-e-Kashmir University of Agricultural Sciences and Technology, Jammu, Jammu and Kashmir, India; 5 Department of Biological Science, Rani Durgavati Vishwavidyalaya, Jabalpur, Madhya Pradesh, India; 6 Department of Animal Genetics Breeding, College of Veterinary Science and Animal husbandry, Nanaji Deshmukh Veterinary Science University, Jabalpur, Madhya Pradesh, India

**Keywords:** Broilers, chTLR4, heterophil:lymphocyte ratio, immunoglobulin G, spleen and bursa of Fabricius and organic trace minerals

## Abstract

**Background and Aim:**

Poultry production is the fastest-growing livestock sector in developing countries. In the poultry diet, trace minerals (zinc [Zn], selenium [Se], and chromium [Cr]) are normally administered in the inorganic form which has been traditionally considered as the most cost-effective and easily available but organic forms of these trace minerals have a higher bioavailability, lower dietary inclusion and cause less environmental pollution as compared to inorganic form. This study aimed to investigate the effect of different concentrations of organic and inorganic forms of trace minerals (Zn, Se, and Cr) supplementation (0-35 days) on expression of chTLR4gene and humoral immune response in broilers.

**Materials and Methods:**

A total of 216 broilers were randomly divided into 12 groups and each group divided into three replicates consisting of six broilers each. T1 (R1, R2, and R3) group was kept as control. T2, T3, and T4 (R1, R2, and R3) groups were supplemented with inorganic form of Zn at 40 mg/kg of feed, organic form of Zn at 40 mg/kg of feed, and 50% organic form of Zn at 20 mg/kg of feed, respectively. T5, T6, and T7 (R1, R2, and R3) groups were supplemented with inorganic form of Se at 0.3 mg/kg of feed, organic form of Se at 0.3 mg/kg of feed, and 50% organic form of Se at 0.15 mg/kg of feed, respectively. T8, T9, and T10 (R1, R2, and R3) groups were supplemented with inorganic form of Cr at 2 mg/kg of feed, organic form of Cr at 2 mg/kg of feed, and 50% organic form of Cr at 1 mg/kg of feed, respectively. T11 and T12 (R1, R2, and R3) groups were supplemented with a combination of all three minerals from inorganic and organic forms, respectively.

**Results:**

Reverse transcriptase-polymerase chain reaction gene expression analysis revealed that in bursa of Fabricius, maximum upregulation of chTLR4 (3.0214 fold) was observed in T6 group, and in spleen, maximum upregulation of chTLR4 (3.2140 fold) was observed in T3 group as compared to control group. On day 35, the maximum plasma immunoglobulin G concentration was observed in organic trace minerals (OTMs) supplemented, whereas the minimum concentration was recorded in control group. On day 28 and 35, the overall mean heterophil:lymphocyte ratio showed a significant difference (p<0.05) between control and OTM supplemented groups. A significantly higher immune organ weight (spleen and bursa of Fabricius) was recorded in OTM supplemented group as compared to control and other supplemented group.

**Conclusion:**

Supplementation of organic form of Zn, Se, and Cr either alone or in combination increase humoral immune response and upregulation of chTLR4 gene expression in bursa and spleen indicates a beneficial effect of OTM in augmentation of immune system in broilers.

## Introduction

Broilers have a higher metabolic rate that makes them more prone to stress which could alter their immunity, productivity, health, or even cause death [1]. Conventionally, inorganic trace minerals (ITMs), zinc (Zn), chromium (Cr), and selenium (Se), are used in broilers’ diet as per the dietary requirement. These ITMs are cost-effective and readily available but are relatively inferior to organic trace minerals (OTMs) due to poor bioavailability. The inorganic minerals chelate with phytic acid complex and reduce their rate of absorption in the gastrointestinal tract and consequently affect the tissue uptake of minerals. In contrast, organic minerals are devoid of free divalent cations for chelation in the intestinal lumen with phytic acid and hence they are differently metabolized, facilitating enhanced absorption. OTMs are highly bioavailable because they have higher retention rate in the body as compared with ITMs and acted as a performance enhancer. Organic minerals could be advantageously incorporated in diet at a lower level than the inorganic sources for realizing higher mineral bioavailability and lower excretion to the environmental [2]. Excessive use of ITMs can result in a detrimental effect on the environment because of their low retention rate and is excreted with the feces [3].

Toll-like receptors (TLRs) which are types of pattern recognition receptors (PRRs) are transmembrane proteins expressed by cells of innate immunity as well as epithelial cells. Modulation of PRR expressed by cells of the innate immune system, including macrophages and dendritic cells would be followed by production of cytokines, some of which are involved in B-cell development and antibody production [4]. TLRs perform a key role as sentinels of the innate immune system. TLRs also facilitate the development of adaptive immune responses. TLR4 was expressed in spleen and bursa of Fabricius [5]. Se plays an important role in innate and adaptive immune cells. Se deficiency can damage both humoral and cellular immunity and deactivate B cells resulting in decreased Ig concentrations [6]. Zn facilitates body biological functions as a catalyst in many enzymes and hormone system that are associated with immunity. Cr is an essential element required for carbohydrate, fat, and protein metabolism. Dietary Cr supplementation increases immunological function in broilers.

This study aimed to determine the effect different concentration of organic and ITMs (Zn, Se, and Cr) supplementation on expression of chTLR4 gene and humoral immune response in broilers.

## Materials and Methods

### Ethical approval

The study was carried out in accordance with the regulations of the Committee for the Purpose of Control and Supervision of Experiments on Animals (CPCSEA) and approved by the Institutional Animal Ethical Committee of College of Veterinary Science and Animal Husbandry, Nanaji Deshmukh Veterinary Science University, Jabalpur vide letter No.118/IAEC/VETY./2018 dated 06/07/2018.

### Study location and period

The research was carried out in the month of February 2020 in the Department of Veterinary Physiology and Biochemistry, College of Veterinary Science and Animal Husbandry, Nanaji Deshmukh Veterinary Science University, Jabalpur, Madhya Pradesh.

### Animals and experimental design

A total of 216 one-day-old commercial broiler (Cobb 500) male chicks were randomly divided into 12 groups and each group was consisting of 18 broilers in three replicates. Number of broilers per treatment was determined by total number of broilers divided by number of treatments. Number of broilers per replicate was determined by total number of broiler per treatment divided by number of replicates. Each replicate consists of six broilers to reduce the sampling error. The experimental design is presented in Table-1. Diets were formulated according to the specification indicated in National Research Centre for starter (0-14 days), grower (15-21 days), and finisher (22-35 days) phase [7]. Feed-grade sulfate salts of Mn, Zn, Fe, and Cu were used, while Se and Cr were in the form of selenite (sodium selenite) and dichromate (potassium dichromate), respectively in the control diet. Organic form of Se (0.30 mg/kg), Cr (2 mg/kg), or Zn (40 mg/kg) was supplemented in treatment groups. The concentration of the Se, Cr, and Zn was taken from previous work conducted by Patel *et al*. [8]. The organic forms of Se, Zn, and Cr (Sel-Plex 2000, Bioplex Zn, and Biochrome, respectively) were a generous gift from Alltech Biotechnology Pvt. Ltd., Bengaluru, India. Concentrations of Zn, Se, and Cr in the above organic TM premixes were 15, 0.2, and 0.1%, respectively (Table-2). All the organic and ITMs were weighed separately in calculated dose and mixed in small quantity of feed then this mixed feed was spread over in total feed allotted to the treatment group and was again mixed properly for uniform mixing of the trace minerals in the feed. All the trace minerals were gradually mixed in the feed using graded dilution manner. Broilers were kept in closed ventilated system for 35 days during the experimental period. The birds were maintained in the battery cage system in a well-ventilated room in the Poultry Experimental Unit at college. The battery brooders were cleaned, washed, and disinfected by blow lamping and whole house was fumigated using formaldehyde and potassium permanganate 4 days before start of the experiment. Feeders and waterers were carefully cleaned with detergent. The feed was offered *ad libitum* in linear chick’s and grower feeders. Aluminum plates of appropriate size and small tin boxes were used in each cage to offer water during early weeks. Due care was taken so that the chicks reach the feeder and waterers in the 1^st^ week of age. Later on, large-size feeders and waterers were attached to each cage in the opposite direction. All-mash system of feeding was practiced during the experiment. Fresh and clean drinking water was made available to birds all the time. Thus, during the entire period of study, uniform conditions of housing, brooding, feeding, and watering were maintained for all the groups of the experiment.

**Table-1:** Experimental design.

Groups	Replicates	No. of broilers	Treatments
T1 (n=18)	T1 R1 T1 R2 T1 R3	6 6 6	Basal diet
T2 (n=18)	T2 R1 T2 R2 T2R3	6 6 6	Basal diet+inorganic zinc at 40 mg/kg of feed
T3 (n=18)	T3 R1 T3 R2 T3R3	6 6 6	Basal diet+organic zinc at 40 mg/kg of feed
T4 (n=18)	T4 R1 T4 R2 T4R3	6 6 6	Basal diet+organic zinc at 20 mg/kg of feed
T5 (n=18)	T5 R1 T5 R2 T5 R3	6 6 6	Basal diet+inorganic selenium at 0.30 mg/kg of feed
T6 (n=18)	T6 R1 T6 R2 T6 R3	6 6 6	Basal diet+organic selenium at 0.30 mg/kg of feed
T7 (n=18)	T7 R1 T7 R2 T7 R3	6 6 6	Basal diet+organic selenium at 0.15 mg/kg of feed
T8 (n=18)	T8 R1 T8 R2 T8 R3	6 6 6	Basal diet+inorganic chromium at 2 mg/kg of feed
T9 (n=18)	T9 R1 T9 R2 T9 R3	6 6 6	Basal diet+organic chromium at 2 mg/kg of feed
T10 (n=18)	T10 R1 T10 R2 T10 R3	6 6 6	Basal diet+organic chromium at 1 mg/kg of feed
T11 (n=18)	T11 R1 T11 R2 T11 R3	6 6 6	Basal diet+inorganic zinc at 40 mg/kg of feed+inorganic selenium at 0.30 mg/kg of feed+inorganic chromium at 2 mg/kg of feed
T12 (n=18)	T12 R1 T12 R2 T12 R3	6 6 6	Basal diet+organic zinc at 40 mg/kg of feed+organic selenium at 0.30 mg/kg of feed+organic chromium at 2 mg/kg of feed

**Table-2:** Ingredients and composition of broiler ration.

Ingredients	Starter %	Finisher %
Maize	43.36	57.30
Soybean meal	43.90	33.10
Soybean oil	08.74	05.61
Common salt	00.40	00.40
DL-methionine	0.185	0.175
DI-calcium phosphate	01.80	01.80
Limestone powder	01.37	01.37
Supplements (vitamins supplement and feed additives)	0.245	0.245

*Trace mineral premix: Mn – 55, I – 0.4, Fe – 56, and Cu – 4 mg/kg ,** vitamin premix: Vitamin A – 8250 IU, Vitamin D_3_ – 1200 IU, Vitamin K – 1 mg, Vitamin E – 40 IU, Vitamin B1 – 2 mg, Vitamin B_2_ – 4 mg, Vitamin B_12_ – 10 mg, percent of values specified by National Register of Citizens, 1994, *** calculated, CP – 23% (0-3 weeks) and 20% (3-5 weeks) ME (weekly) – 1^st^ weeks – (432 Kcal/bird), 2^nd^ weeks – (928 Kcal/bird), 3^rd^ weeks (1558 Kcal/bird), 4^th^ weeks (2256 Kcal/bird), and 5^th^ weeks – 3075 Kcal/bird in male

### chTLR4 genes expression analysis studies

Two broilers per replicate were sacrificed on day 35 of the experiment and organs, including bursa of Fabricius and spleen were collected aseptically in RNA later (stabilize and protect RNA with immediate RNase inactivation) for RNA extraction and isolation. These broilers were sacrificed as per the appropriate standard procedure. Total RNA was isolated from the bursa of Fabricius and spleen using standard protocol using TRIzol method. The detailed protocol followed by taking 50 mg of fresh tissue samples, which were homogenized with 1 mL TRIzol reagent. The homogenized samples were then incubated at room temperature (25ºC) for 5 min. Two hundred microliters of chloroform were added per mL of TRIzol reagent used and mixed vigorously for 15 s and incubated at room temperature (25ºC) for 10-15 min. The resultant mixture was then centrifuged at 12,000×g for 15 min at 4°C. The upper aqueous phase was transferred to a fresh 2 mL microcentrifuge tube and then 500 µL of chilled 2-propanol added for precipitation of RNA. The resultant mixture was then incubated at −20°C for 20 min and centrifuged at 12,000×g for 10 min at 4°C. The RNA precipitates in a pellet form at the bottom of the tube. The supernatant was then carefully removed and RNA pellet washed by adding 1 mL of 75% ethanol. It was followed by gentle tapping and centrifugation at 7500×g for 5 min at 4°C. The supernatant was then removed and RNA pellet was air-dried in an RNase-free environment. RNA pellet was then dissolved in 50 µL of nuclease-free water by repeated pipetting with a micropipette. The purity of the total RNA was confirmed by considering the ratios of optical density values at 260 and 280 nm between 1.9 and 2.0. The integrity of RNA was checked on 1.0% agarose gel using ×1 TBE as electrophoresis buffer. The RNA samples showing contamination with DNA were incubated with RNase-free DNase-1 (MBI Fermentas, USA) at 37°C for 30 min (at 1 U for 1 µg total RNA). The DNase was subsequently inactivated by incubation at 65°C for 20 min after adding the 25 mM EDTA (at 1 µL for 1 µg total RNA). The purity and concentration of DNase-treated total RNA sample was determined using a Nanodrop spectrophotometer (ND 2000, Thermo Fisher Inc., USA).

The concentration of RNA was checked by Nanodrop 2000 (Thermo Fisher Inc.) before the preparation of first-strand cDNA. Prepared cDNA was stored at −20°C and further used for chTLR4 gene expression studies. Expression of chTLR4 gene was quantified by gene-specific primer pairs using real-time polymerase chain reaction (PCR). β-actin was used as a housekeeping gene. The purity and concentration of the total RNA was assessed using a Nanodrop spectrophotometer (ND 2000, Thermo Fisher Inc.).

### First-strand cDNA synthesis

The first-strand cDNA was synthesized using RevertAid™ First-Strand cDNA Synthesis kit (MBI Fermentas Vilnius, Lithuania). Primers for chTLR4 and β-actin (β-actin; used as housekeeping gene) were adopted [2]. The sequence of gene-specific primers for chTLR4 and β-actin is mentioned in Table-3.

**Table-3:** Sequence of gene-specific primers for chTLR4 and β-actin.

S. No.	Gene	Primers	Annealing temp.	GenBank accession number
1.	chTLR4	F: AGTCTGAAATTGCTGAGCTCAAAT R: GCGACGTTAAGCCATGGAAG	59°C	AY064697
2.	β-actin	F: CAACACAGTGCTGTCTGGTGGTA R: ATCGTACTCCTGCTTGCTGATCC	60°C	X00182

### PCR reaction mixture

ReadyMix Taq PCR Reaction Mix with MgCl_2_ (Sigma-Aldrich, USA) was used to prepare PCR reaction mixture. In a PCR tube, 25 µL reaction mixtures were prepared as per Table-4.

**Table-4:** Component of the PCR reaction mixture.

2×ReadyMix Taq PCR Reagent Mix	12.5 µL
Forward primer (10 pM)	1.0 µL
Reverse primer (10 pM)	1.0 µL
cDNA	1.0 µL
Nuclease-free water	9.5 µL
Total volume	25 µL

Mix gently and briefly centrifuge to collect all components to the bottom of the tube. The PCR tubes with all the components were then transferred to a thermal cycler (ABI Veriti, Inc., USA). The PCR protocol designed for 35 cycles is mentioned in Table-5, and it was kept same for both the primers.

**Table-5:** The PCR protocol of chTLR4 and β-actin gene.

S. No.	Steps		chTLR4	β-actin
1.	Initial denaturation	Temperature	94°C	94°C
		Time	10 min	10 min
2.	Denaturation	Temperature	94°C	94°C
		Time	1 min	1 min
3.	Annealing	Temperature	59°C	60°C
		Time	45 s	45 s
4.	Extension	Temperature	72°C	72°C
		Time	1 min	1 min
5.	Final extension	Temperature	72°C	72°C
		Time	10 min	10 min
6.	Hold	Temperature	4°C	4°C
		Time	∞	∞

### Agarose gel electrophoresis of PCR products

The PCR products were tested for amplification of specific genes by agarose gel electrophoresis using 2.0% agarose gel in ×1 Tris-acetate EDTA Buffer (Sigma-Aldrich). A total volume of 60 mL of 2.0% agarose (Sigma-Aldrich) was prepared in ×1 Tris-acetate EDTA Buffer and placed in microwave oven until melted. Molten agarose was allowed to cool to about 55°C and ethidium bromide was added to give a final concentration of 0.5 µg/mL. The gel was poured onto electrophoresis through fitted with a comb. The gel was allowed to set on a flat surface for about 15 min. Electrophoresis was placed in an electrophoresis tank filled with ×1 Tris-acetate EDTA Buffer. Samples were prepared on a parafilm by mixing 2 µL of Gel Loading Buffer (Sigma-Aldrich) and 8 µL of PCR products were loaded in parallel with 100 bp ladder (Direct load PCR 100 bp low ladder, Sigma-Aldrich). Electrophoresis was done at 70 volts for 10 min, then at 50 volts for 2 h. Gel was viewed under an ultraviolet transilluminator and photographed with gel documentation system (*BIO RAD* Gel Doc EZ Images, USA) for future analysis.

### Quantitative reverse transcriptase-PCR (qRT-PCR/real-time PCR)

The relative expression of gene-specific mRNA was quantified by qRT-PCR/real-time PCR employing SYBR green chemistry using a real-time PCR system (7500 RT-PCR, Thermo Fisher, Inc). All reactions were performed in nuclease-free 8 tube strips with optically clear flat caps (ABI, Thermo Fisher Inc). For each sample, a dissociation curve (melting curve) was generated after the completion of amplification to ascertain the specificity of amplification. A negative control containing all the ingredients except cDNA template ­(non-template control) was set up invariably for each master mix made for conducting the reactions. The results were expressed as C_T_ values of target and reference genes in test (treatment) and control (calibrator) samples.

### Real-time PCR reaction mixture

The component of real-time PCR reaction mixture is mentioned in Table-6.

**Table-6:** Component of real-time PCR reaction mixture.

2× Power SYBR Green q PCR master mix	10 µL
Forward primer (10 pM)	0.1 µL
Reverse primer (10 pM)	0.1 µL
cDNA	01 µL
Nuclease-free water	8.8 µL
Total volume	20 µL

### Real-time PCR reaction protocol

PCR cycling conditions were initial denaturation of 94°C for 10 min, followed by 40 cycles of denaturation 94°C for 1 min for all genes; annealing temperature for chTLR4 (59°C), and β-actin (60°C) for 45 s and extension 72°C for 1 min, final extension 72°C for 10 min, and hold at 4°C.

### Relative quantification

Comparative C_T_ method [9] was used for relative expression of target gene in the test sample (treatment) relative to that of control sample (calibrator). The mRNA expression of target gene in test sample was expressed as “n-fold up/down-regulation” in relation to control sample. For estimation of relative expression of target gene by the comparative C_T_ method, C_T_ values of target gene in test and control sample were adjusted to the C_T_ values of a housekeeping gene (endogenous/internal control). In the present study, chTLR4 was the target gene, whereas β-actin was taken as a housekeeping gene. The C_T_ for the target gene (chTLR4) and the C_T_ for the housekeeping gene (β-actin) were determined for each test sample and the control sample. The relative expression of target genes was estimated in terms of fold change in mRNA expression, using the following formula:

Fold change in expression of target gene = 2^−ΔΔC^_T_

where,

∆∆ C_T_ = ∆ C_T test_−∆C_T control/calibrator_

∆ C_T test_ = C_T target gene_−C_T reference gene_ (In test/treatment group)

∆C_T control/calibrator_ = C_T target gene_−C_T reference gene_ (In control/calibrator group)

where,

CT target gene= Mean of the cycle threshold (CT) value of the gene being tested

CTreference gene= Mean of the CT value of the housekeeping gene β-actin.

### Humoral immune responses

#### Chicken plasma immunoglobulin G (IgG) estimation

The 72 samples employed for the quantitative estimation of chicken IgG levels were the sandwich ELISA technique.

#### Heterophil/lymphocyte (H:L) ratio

H:L was counted for six samples per treatment by staining the blood smears by Leishman’s stain and manually counting as per the method outlined [10]. At least 100 cells were counted.

#### Immune organ weight of broilers

After 35 days of age, two birds per replicate with the average body weight per group were sacrificed by cervical dislocation. Immediately after bleeding from the jugular vein, several organs, including the bursa of Fabricius and spleen were harvested and weighed individually.

### Statistical analysis

Statistical analysis was done with the help of IBM SPSS-24 software (IBM, USA), using one-way analysis of variance with Duncan *post hoc* multiple comparisons and descriptive statistics (p≤0.05).

## Results and Discussion

### RT-PCR expression analysis of TLR4 in bursa of Fabricius and spleen of broiler

**Figure-1:**
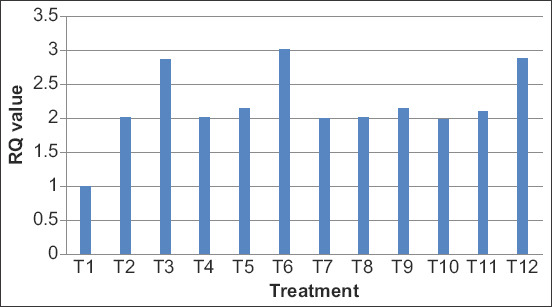
Expression profiling of chTLR4 gene in bursa of Fabricius of different treatment groups in broiler.

**Figure-2:**
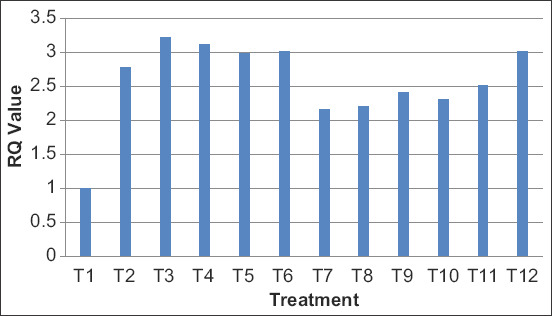
Expression profiling of chTLR4 gene in spleen of different treatment groups in broilers.

On day 35, the mRNA expression levels of TLR4 gene in bursa of Fabricius of broiler birds have been presented in terms of fold change expression in Table-7 and Figure-1 and 2. RT-PCR expression analysis of chTLR4 gene revealed that in bursa of Fabricius, maximum upregulation (3.0214 fold) was observed in T6 group, followed by T12 (2.8865 fold) group, and in spleen, maximum upregulation (3.2140) was observed in T3 group as compared to control group. Significantly higher expression of chTLR4 *gene* in the bursa of Fabricius and spleen was observed (p<0.05) in yeast-derived molecules treatment as compared with the control [11] which is in agreement with the present findings. Similar findings were also reported in his study to determine the change in chTLR4 mRNA expression in the liver, kidney spleen, heart, and small intestine of broiler chickens under acute heat stress condition. They found that chTLR4 mRNA expression at HS10 in different organs was significantly higher (p<0.001) compared with HS2 and HS5. The acute heat stress modulates the functional responses of the liver, kidney, spleen, heart, and small intestine of broilers by regulating chTLR4 mRNA expression [12]. Activation of TLR results synthesis and release of pro-inflammatory cytokines. The possible reason may be the presence of these cytokines modulates adaptive immunity. The manipulation of gut mineral bioavailability through supplementation of OTM can influence cell and antibody-mediated immune response. The main target for supplementation of OTM is the reduction of local inflammation and limitation of further impairment of immune function [13].

**Table-7:** Comparative gene expression profiling (fold change) of TLR4 gene in different treatment groups of broilers.

Treatment	Bursa of Fabricius	Spleen
T1	1.0000	1.0000
T2	2.0251	2.7741
T3	2.8714	3.2140
T4	2.0124	3.1120
T5	2.1470	2.9841
T6	3.0214	3.0124
T7	1.9985	2.1584
T8	2.0132	2.2014
T9	2.1475	2.4150
T10	1.9856	2.3014
T11	2.1034	2.5210
T12	2.8865	3.0140

In disagreement with the present findings, the effect of yeast-derived carbohydrates on gene expression of chTLR4 was downregulated in YDC treatment compared with control in the ileum [14]. In contrary to our findings, the relative mRNA gene expression of chTLR4 in spleen of Ross broiler chickens was not affected by yeast cell wall and virginiamycin supplementation [15] The possible reason for such an expression may be because of the involvement in leukocyte attraction and directs growth, maturation, and differentiation of many cell types, in addition to enhancing NK cell activity and regulating B-cell functions such as Ig production and class switching [16].

### Chicken plasma IgG concentration

**Figure-3:**
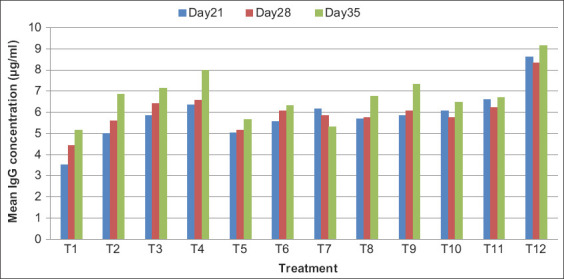
Mean plasma IgG concentration (µg/mL) in broilers at different intervals.

Chicken plasma IgG concentration in broilers is presented in Table-8 and Figure-3. The mean concentration of plasma IgG showed a significant difference (p<0.05) between all the treatment groups. On days 21, 28, and 35, a significantly (p<0.05) higher concentration of plasma IgG was reported in T12 group as compared to T1. The mean concentration of plasma IgG showed a non-significant difference between all the groups. However, the maximum concentration of plasma IgG was found in T12 group, whereas the minimum concentration of plasma IgG was found in T1 group during the entire investigation period. Similar results were reported where supplementation of organic Zn, Se, and Cr did not significantly affect the antibody titer of Newcastle diseases vaccine [17]. Contrary to these findings, organic mineral forms resulted in significantly higher titers of both IgM and IgG as compared to inorganic mineral forms in heat-stressed broiler chickens [18].

**Table-8:** Mean plasma IgG concentration (µg/mL) in broilers at different intervals.

Treatment	Day 21	Day 28	Day 35
T1	3.52^c^±0.27	4.42^b^±0.32	5.15^c^±0.27
T2	5.00^bc^±0.48	5.60^b^±0.43	6.87^bc^±0.43
T3	5.85^b^±0.45	6.42^ab^±0.35	7.15^abc^±0.33
T4	6.35^b^±0.81	6.58^ab^±0.71	7.98^ab^±0.40
T5	5.02^bc^±0.27	5.15^b^±0.29	5.65^c^±0.60
T6	5.58^bc^±0.55	6.08^b^±0.54	6.32^bc^±0.87
T7	6.15^bc^±0.65	5.85^b^±0.44	5.31^c^±0.73
T8	5.68^bc^±0.88	5.75^b^±0.97	6.75^bc^±0.78
T9	5.85^bc^±0.39	6.08^b^±0.63	7.32^abc^±0.53
T10	6.07^b^±1.15	5.75^b^±0.97	6.48^bc^±1.00
T11	6.62^b^±1.07	6.23^b^±0.10	6.70^bc^±1.12
T12	8.61^a^±0.63	8.33^a^±0.63	9.16^a^±0.22

Means bearing different superscripts within same column differ significantly (p<0.05)

The lack of immune responses in the present study might be due to variation in the dose of the minerals used. Furthermore, in many reports, it has been observed that stress causes suppression of antibody production in young chickens. This reduction could be indirectly due to an increase in inflammatory cytokines under stress, which stimulates the hypothalamic production of corticotrophin-releasing factor. Corticotrophin-releasing factor is known to increase adrenocorticotropic hormone (ACTH) from the pituitary, which, in turn, stimulates corticosterone production from the adrenal gland. It has been well documented that corticosterone inhibits antibody production. The higher response observed might be due to use of relatively higher concentrations of Zn (up to 181 mg/kg) as compared to the levels used in the present study (20 and 40 mg Zn/kg). The data obtained in the present investigation showed the same pattern as the higher requirement of Zn (120 and 200 mg/kg) for augmentation of humoral immune response in chickens [19].

Dietary Se (inorganic Se) could improve immunity and significantly enhance the synthesis of IgA and IgG in broilers. Se supplementation of both inorganic and bacterial organic sources improved the bird’s immunity through IgA, IgG, and IgM elevation on day 42, but on day 21, bacterial organic Se showed significantly highest IgG levels as compared to sodium selenite and without Se supplemented groups [20]. Supplementation of 90 mg Zn per kilogram of feed as Zinc-Glycine chelate improved immunoglobulins levels (IgA, IgM, and IgG) in broilers [21].

Immune activity in poultry is influenced by micro- and macronutrients, particularly Zn, which is included in poultry diets as feed additives. Zn has been shown to modulate the body’s immune response. In particular, Zn increases the activation and proliferation of lymphocytes, mainly T and natural killer (NK) cells, and stimulates cellular defense mechanisms. The immunomodulatory effect of Zn also results in an increase in the activity of thymocytes, macrophages, and heterophils, as well as increased antibody production, which enhances the potential of the humoral response [22].

### H:L ratio

**Figure-4:**
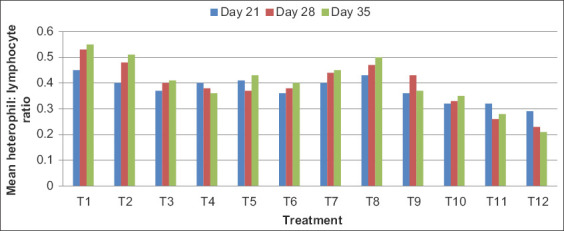
Mean heterophil:lymphocyte ratio in broilers at different intervals.

The mean H:L ratio of broilers is presented in Table-9 and Figure-4. The mean H:L ratio showed a non-significant difference (p<0.05) among all the groups on day 21, whereas a significant difference (p<0.05) was found between all the groups on days 28 and 35. On days 21, 28, and 35, the highest H:L ratio was observed in T1 and the lowest H:L ratio was observed in T12 group. The mean H:L ratio showed a significant difference (p<0.05) between all the groups. A higher H:L ratio was observed in control group which declined with the supplementation of OTM.

**Table-9:** Mean heterophil: lymphocyte ratio in broilers at different intervals.

Treatment	Day 21	Day 28	Day 35
T1	0.45±0.69	0.53^a^±0.06	0.55^a^±0. 07
T2	0.40±0.03	0.48^ab^±0.05	0.51^ab^±0.06
T3	0.37±0.07	0.40^abcd^±0.06	0.41^abc^±0.05
T4	0.40±0.03	0.38^abcd^±0.03	0.36^bcd^±0.03
T5	0.41±0.02	0.37^abcd^±0.05	0.43^abc^±0.02
T6	0.36±0.07	0.38^abcd^±0.03	0.40^abc^±0.05
T7	0.40±0.08	0.44^abc^±0.07	0.45^abc^±0.08
T8	0.43±0.06	0.47^ab^±0.06	0.50^ab^±0.02
T9	0.36±0.06	0.43^abc^±0.07	0.37^abcd^±0.01
T10	0.32±0.04	0.33^bcd^±0.03	0.35^bcd^±0.03
T11	0.32±0.03	0.26^cd^±0.02	0.28^cd^±0.04
T12	0.29±0.03	0.23^d^±0.04	0.21^d^±0.01

Means bearing different superscripts within same column differ significantly (p<0.05).

In the current study, an increase in lymphocytes and decrease in heterophils count and consequently decrease in H:L ratios in broiler chicks fed with different level and forms of organic Zn, Se, and Cr were observed. Similar findings were also reported that H:L ratio was significantly decreased in heat-stressed broiler chicks fed 1000 and 1500 μg kg^−1^ Cr picolinate [23].

Heterophils are particularly sensitive to stressors such as ACTH. The probable reason for increase in lymphocytes and decrease in heterophils count and decrease in H:L ratio by organic Zn, Se, and Cr supplementation in broilers in this study may be due to a reduction in glucocorticoid secretions or higher production of interleukin-2.

The present finding also correlates with that there was a decrease in H:L ratios in broiler chickens fed 800 ppb Cr on 21 and 42 days of experimentation [24]. Significant decrease in H:L ratio by feeding Zn sulfate and Cr picolinate to Japanese quails [25]. H:L ratio was decreased with Cr propionate dosage, which was suggestive of reduced stress levels in broilers [26]. H/L ratio was higher in the OTM treatment group as compared to control (p<0.05) [2], which is in disagreement with the present findings.

### Immune organ weight of broilers

**Figure-5:**
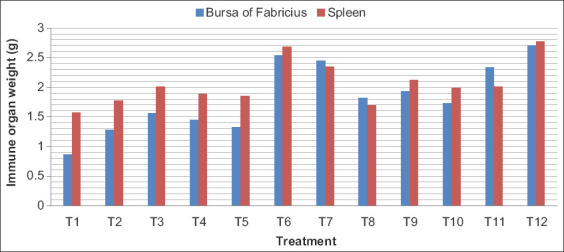
Immune organ weight (g) of broilers.

The weight of bursa of Fabricius and spleen is presented in Table-10 and Figure-5. Maximum weight of bursa of Fabricius and spleen was found in T12 group and minimum was found in T1 group. In accordance with the present finding that supplementation of 90 mg Zn per kilogram of feed as Zinc-Glycine chelate increased the immune organs index in the broilers [21]. Different levels and sources of Zn, copper, and manganese increased the relative weight of spleen and bursa of Fabricius [27]. Supplemental dietary Cr, particularly at the level of 1200 ppb of Cr L-methionine, improved body weight gain and weight of the lymphoid organs, but not significantly [28], which is in agreement to the present findings.

**Table-10:** Immune organ weight (g) of broilers.

Treatment	Bursa of Fabricius	Spleen
T1	0.86	1.57
T2	1.28	1.77
T3	1.56	2.01
T4	1.45	1.89
T5	1.32	1.85
T6	2.53	2.68
T7	2.45	2.35
T8	1.82	1.69
T9	1.93	2.12
T10	1.73	1.98
T11	2.34	2.01
T12	2.70	2.77

Contrary to these findings that the dietary supplementation of organic Cr in commercial broiler chickens has no effect on the relative mass of lymphoid organs such as bursa, spleen, and thymus by supplementing graded concentrations (0, 100, 200, 300, or 400 μg/kg diet) of organic chromium [29]. Supplementation with CrMet had a non-significant effect on the weight of lymphoid organs of broilers in different groups [24]. Supplementation of chromium propionate at different dosage levels (100 to 3,200 µg/kg of elemental chromium in feed) had no significant effect on lymphoid organ weights of male broiler chickens (Cobb 400) for the study period of 42 days [26].

## Conclusion

Supplementation of organic form of Zn, Se, and Cr either alone or in combination increase humoral immune response and upregulation of chTLR4 gene expression in bursa and spleen indicates a beneficial effect of OTM in augmentation of the immune system in broilers.

## Authors’ Contributions

AM and PP designed and guided the research steps. AKJ conducted the experimental trial and research work and collected the data. APS, TRC, and AAS guided the storage of the collected samples and processing of samples. RV conducted the statistical analysis of the data and created the graphs. All authors read and approved the final manuscript.

## References

[1] Echeverry H, Yitbarek A, Munyaka P, Alizadeh M, Cleaver A, Camelo-Jaimes G, Wang P.O.K, Rodriguez-Lecompte J.C (2016). Organic trace mineral supplementation enhances local and systemic innate immune responses and modulates oxidative stress in broiler chickens. Poult. Sci.

[2] Aksu D, Aksu T, Ozsoy B, Baytok E (2010). The effects of replacing inorganic with a lower level of organically complexed minerals (Cu, Zn and Mn) in broiler diets on lipid peroxidation and antioxidant. Asian Australas. J. Anim. Sci.

[3] Lukasz J, Agnieszka M, Zbigniew G, Malgorzata K, Marcin K (2017). The effect of feed supplementation with zinc chelate and zinc sulphate on selected humoral and cell-mediated immune parameters and cytokine concentration in broiler chickens. Res. Vet. Sci.

[4] Hirayama D, Lida T, Nakase H (2018). The phagocytic function of macrophage-enforcing innate immunity and tissue homeostasis. Int. J. Mol. Sci.

[5] Kawasaki T, Kawai T (2014). Toll-like receptor signaling pathways. Front. Immunol.

[6] Woods S.L, Sobolewska S, Rose S.P, Whiting M, Blanchard A, Ionescu C, Bravo D, Pirgozliev V (2020). Effect of feeding different sources of selenium on growth performance and antioxidant status of broilers. Br. Poult. Sci.

[7] NRC (1994). Nutrient Requirement of Poultry.

[8] Patel P, Mishra A, Singh A.P, Jain A.K, Kumari M, Sheikh A.A (2019). Supplementation of inorganic and organic forms of zinc, selenium and chromium on immune responses in broiler chicken. Int. J. Chem. Stud.

[9] Livak K.J, Schmittgen T.D (2001). Analysis of relative gene expression data using real-time quantitative PCR and the 2(-Delta Delta C(T)). Methods.

[10] Feldman B.F, Jain N.C (2002). Schalm's Veterinary Haematology.

[11] Yitbarek A, Rodriguez-Lecompte J.C, Echeverry H.M, Munyaka P, Barjesteh N, Sharif S, Camelo-Jaimes G (2013). Performance, histomorphology, and Toll-like receptor, chemokine and cytokine profile locally and systemically in broiler chickens fed diets supplemented with yeast-derived macromolecules. Poult. Sci.

[12] Huang S (2017). Upregulation of TLR4 mRNA expression levels in broiler chickens under acute heat stress. Braz. J. Poult. Sci.

[13] Nawab A, An L, Wu J (2019). Chicken toll-like receptors and their significance in immune response and disease resistance. Int. Rev. Immunol.

[14] Munyaka P, Echeverry H, Yitbarek A, Camelo-Jaimes G, Sharif S, Guenter W, House J.D, Rodriguez-Lecompte J.C (2012). Local and systemic innate immunity in broiler chickens supplemented with yeast-derived carbohydrates. Poult. Sci.

[15] Alizadeh M, Rodriguez-Lecompte J.C, Yitbarek A, Sharif S, Slominski B.A (2016). Effect of yeast-derived products on systemic innate immune response of broiler chickens following a lipopolysaccharide challenge. Poult. Sci.

[16] Schroder K, Sweet M.J, Hume D.A (2006). Signal integration between IFN-γand TLR signaling pathways in macrophages. Immunobiology.

[17] Rao S.V.R, Prakash B, Raju M.V.L, Panda A.K, Kumari R.K, Reddy E.P.K (2016). Effect of supplementing organic forms of zinc, selenium and chromium on performance, antioxidant and immune responses in broiler chicken reared in tropical summer. Biol. Trace Elem. Res.

[18] Ghazi S, Habibian M, Moeini M.M, Abdolmohammadi A.R (2012). Effects of different levels of organic and inorganic chromium on growth performance and immunocompetence of broilers under heat stress. Biol. Trace Elem. Res.

[19] Sajadifar S, Miranzadeh H, Moazeni M (2013). Effect of zinc on humoral and cell-mediated immunity of broilers vaccinated against coccidiosis. Iran. J. Parasitol.

[20] Khan M.Z.I, Akter S.H, Islam M.N, Karim M.R, Islam M.R, Kon Y (2008). The effect of selenium and Vitamin E on the lymphocytes and immunoglobulin-containing plasma cells in the lymphoid organ and mucosa-associated lymphatic tissues of broiler chickens. Anat. Histol. Embryol.

[21] Feng J, Ma W.Q, Niu H, Wu X, Wang Y (2010). Effects of zinc glycine chelate on growth, hematological, and immunological characteristics in broilers. Biol. Trace Elem. Res.

[22] Jarosz L, Marek A, Gradzki Z (2019). Effect of zinc sulfate and zinc glycine chelate on concentrations of acute-phase proteins in chicken serum and liver tissue. Biol. Trace Elem. Res.

[23] Toghyani M, Khodami A, Gheisari A.A (2008). Effect of organic chromium supplementation on meat quality of heat-stressed broiler chicken. Am. J. Anim. Vet. Sci.

[24] Ebrahimzadeh S.K, Farhoomand P, Noori K (2012). Immune response of broiler chickens fed diets supplemented with different levels of chromium methionine under heat stress conditions. Asian Australas. J. Anim. Sci.

[25] Rouhalamini S.M, Salarmoini M, Asadi-Karam G (2014). Effect of zinc sulfate and organic chromium supplementation on the performance, meat quality and immune response of Japanese quails under heat stress conditions. Poult. Sci. J.

[26] Rajalekshmi M, Sugumar C, Chirakkal H, Ramarao S.V (2014). Influence of chromium propionate on the carcass characteristics and immune response of commercial broiler birds under normal rearing conditions. Poult. Sci.

[27] Gheisari A.A, Rahimi-Fathkoohi A, Toghyani M, Gheisari M.M (2011). Influence of feeding diets supplemented with different levels and sources of zinc, copper and manganese on the mineral concentrations in tibia and performance of broiler chickens. Asian J. Anim. Vet. Adv.

[28] Bahrami A, Moeini M.M, Ghazi S.H, Targhibi M.R (2012). The effect of different levels of organic and inorganic chromium supplementation on immune function of broiler chicken under heat-stress conditions. J. Appl. Poult. Res.

[29] Rao S.V, Raju M.V, Panda A.K, Poonam N.S, Murthy O.K, Sunder G.S (2012). Effect of dietary supplementation of organic chromium on performance, carcass traits, oxidative parameters and immune responses in commercial broiler chickens. Biol. Trace Elem. Res.

